# Distribution and clinicopathological characteristics of G-CSF expression in tumor cells and stromal cells in upper tract urothelial carcinoma

**DOI:** 10.1007/s00432-024-06045-1

**Published:** 2024-12-30

**Authors:** Go Kobayashi, Yohei Sekino, Hikaru Nakahara, Kohei Kobatake, Keisuke Goto, Tetsutaro Hayashi, Kazuhiro Sentani, Nobuyuki Hinata

**Affiliations:** 1 Laboratory of Molecular Pathology, Department of Molecular Biosciences, Radiation Effects Research Foundation, Hiroshima, Japan; 2 Department of Molecular Pathology, Graduate School of Biomedical and Health Sciences, Hiroshima University, Hiroshima, Japan; 3 Department of Urology, Graduate School of Biomedical and Health Sciences, Hiroshima University, 1-2-3 Kasumi, Minami-ku, Hiroshima, 734-8551 Japan; 4 Department of Clinical and Molecular Genetics, Graduate School of Biomedical and Health Sciences, Hiroshima University, Hiroshima, Japan

**Keywords:** Clinicopathological significance, G-CSF, Immunohistochemistry, Prognostic marker, Upper tract urothelial carcinoma

## Abstract

**Background:**

Urothelial carcinoma (UC) is a common type of malignant disease; however, the diagnostic and prognostic markers of upper urinary tract urothelial cancer (UTUC) remain poorly understood because of its rarity.

**Methods:**

To clarify the clinicopathological significance of granulocyte-colony stimulating factor (G-CSF) in UTUC, we analyzed the expression and distribution of G-CSF in 112 upper tract urothelial carcinoma (UTUC) samples with immunohistochemistry.

**Results:**

In normal urothelium, G-CSF expression was weak or absent, whereas high expression of G-CSF was observed in UTUC tissues, both in tumor cells (TCs) and stromal cells (SCs). G-CSF expression in the TCs and SCs was associated with nodular/flat morphology, high grade, advanced T stage, and lymphovascular invasion in UTUC. G-CSF expression in SCs was associated with poor prognosis and was an independent prognostic factor. Public data showed that G-CSF expression was also associated with decreased progression-free survival and disease-specific survival. A prognostic model was constructed by incorporating the presence or absence of G-CSF expression along with clinicopathologic factors, which allowed for a more accurate prediction of poor prognosis. We further showed that G-CSF expression was associated with a high Ki-67 labeling index and with PD-L1, HER2, and p53 expression in UTUC.

**Conclusion:**

G-CSF expression in TCs and SCs may play a crucial role in UTUC tumor progression. Notably, stromal G-CSF expression showed significant prognostic value, even when compared to major clinicopathological factors, suggesting that the evaluation of G-CSF expression may contribute to clinical decision-making in patients with UTUC.

**Supplementary Information:**

The online version contains supplementary material available at 10.1007/s00432-024-06045-1.

## Introduction

Urothelial carcinoma (UC) is a common malignancy. Most cases of UC are of urinary bladder UC, and upper tract UC (UTUC) is relatively rare, accounting for approximately 5–10% of all urothelial tumors (Rouprêt et al. 2018). The prognosis of advanced UC remains poor: 5-year cancer-specific survival (CSS) in patients with pT4 disease is < 10% (Rouprêt et al. 2018; Leow et al. 2016; Abouassaly et al. 2010). In contrast to bladder UC, UTUCs present as an invasive disease at diagnosis in 60% of cases and have a poor prognosis (Kolawa et al. 2023). Thus, prognostic predictors are important for guiding therapeutic options and surveillance strategies in UTUC (Favaretto et al. 2018). Various clinicopathological parameters including stage, grade, concomitant carcinoma in situ (CIS), and lympho-vascular and venous invasion have been shown to be prognostic factors (Rouprêt et al. 2018; Leow et al. 2016; Li et al. 2020; Margulis et al. 2009). However, their predictive accuracy remains limited due to individual variation. Despite this, little is known about the efficacy of prognostic markers and therapeutic targets in UTUC. Clinical decision-making involving UTUC requires the identification of useful prognostic biomarkers and novel therapeutic targets.

Recent reports indicate that several biomarkers (e.g., HER2, EGFR, PD-L1, CD8, claspin, MUC1, ANXA10, TUBB3, and MCM4) have prognostic significance in UTUC (Favaretto et al. 2018; Chen et al. 2021b; Hagiwara et al. 2016; Kobayashi et al. 2021, 2022a, b, 2023a, b). Although biomarkers often focus on the expression in tumor cells (TCs), the molecular characteristics of tumor stromal cells (SCs) have important implications for tumorigenesis and tumor progression. The tumor microenvironment (TME) is well known to greatly influence tumor development and progression (Wang et al. 2023). However, reports focusing on the TME in UTUC are limited. Tumor-associated neutrophils (TANs) play a crucial role in tumor development and progression within the TME (Yan et al. 2022). Recent studies have shown that granulocyte-colony stimulating factor (G-CSF) derived from TANs induces their activation and differentiation (Karagiannidis et al. 2021; He et al. 2022). G-CSF, also known as colony-stimulating factor 3 (CSF3), is a critical regulator of the proliferation, differentiation, and survival of granulocytes (Karagiannidis et al. 2021; Dwivedi and Greis 2017). G-CSF production by malignant tumors has been reported in several cancers, such as lung, breast, and cervical cancer, and has been associated with poor clinical outcomes (Karagiannidis et al. 2021). However, to our knowledge, the clinicopathological significance of G-CSF in UC remains largely unknown. Moreover, previous reports investigating G-CSF expression in various cancers have focused primarily on TCs, and the distribution and clinicopathological significance of G-CSF in tumor SCs are still poorly understood.

In the present study, we clarified the distribution and clinicopathological significance of G-CSF expression in UC for the first time, to our knowledge, using surgical UTUC tissue specimens. We chose to analyze UTUC samples because this approach allows comparison of the correlations between molecular expression and clinicopathological factors across various stages of UTUC treated by radical nephroureterectomy, without the influence of modifications such as transurethral resection. We also evaluated the association between G-CSF expression and representative cancer-related molecules.

## Materials and methods

### Tissue samples

The medical records of patients who underwent radical nephroureterectomy for unilateral UTUC at Hiroshima University Hospital between April 1999 and May 2019 were retrospectively reviewed. Patients with neoadjuvant chemotherapy were excluded from this study. Thus, 112 patients (mean age, 71.4 years; standard deviation 10.2 years; male, *n* = 82 [73%]) were included. Pathology specimens were examined and rereviewed for staging according to the 8th edition of the American Joint Committee on Cancer/Union for International Cancer Control (AJCC/UICC) TNM classification (2017). We used the 2004 WHO/ISUP 2-tier grading system to evaluate the tumor grade. During the follow-up period, cancer deaths were observed in 18 (16%) patients, and the study endpoint was CSS. Our follow-up protocol consisted of a urine analysis and chest-abdomen-pelvis computed tomography with or without contrast every 3–6 months for at least five years, according to the preferences of each physician. The final follow-up date was August 1, 2020.

### Immunohistochemistry

Immunohistochemistry was performed on one or two representative tumor blocks, including the tumor center and invading front according to the manufacturer’s protocol. Immunohistochemical analysis was performed using a Dako Envision + Mouse Peroxidase Detection System (Dako Cytomation, Carpinteria, CA). Antigen retrieval was performed by microwave heating in citrate buffer (pH 8.0) for 60 min. Peroxidase activity was blocked with 3% H_2_O_2_-methanol for 5 min, and the sections were incubated with normal goat serum (Dako Cytomation) for 20 min to block nonspecific antibody binding sites. Sections were incubated with a goat polyclonal anti-G-CSF K-15 sc-49,679 antibody (dilution 1:500, Santa Cruz. Biotechnology) for 1 h at room temperature, followed by incubation with Envision + antimouse peroxidase for 1 h. The sections were incubated with DAB Substrate-Chromogen Solution (Dako Cytomation) for 5 min for color reaction and were then counterstained with 0.1% hematoxylin. Negative controls were created by omission of the primary antibody. The expression of G-CSF was evaluated in TCs and SCs, respectively. G-CSF expression in TCs was analyzed using the histoscore (H-score). The percentage of G-CSF expression in SCs was calculated.

The evaluation of Ki-67, PD-L1, CD44v9, HER2, EGFR, FGFR3, p53, GATA3, and CK5/6 was described previously (Kobayashi et al. 2021, 2022a, b, 2023a, b). We defined the following as positive: >20% of cancer cells stained for GATA3, all layers stained for CK5/6, and > 10% of cancer cells stained for CD44v9 and TP53. The expression of HER2, EGFR, and FGFR was scored according to the intensity of antibody staining (0 = no staining, 1 + = weak staining, 2 + = moderate staining, and 3 + = intense staining), and all cases with staining intensity of 3 + or 2 + were defined as positive The expression of PD-L1 was evaluated on the membrane of TCs and tumor-infiltrating lymphocytes (TILs) and was considered positive according to median cutoff values rounded off to the nearest 1% on TCs and TILs, respectively.

### In silico analysis

The expression array data were downloaded from GEO and Array Express under accession number GSE166912 (Karanović et al. 2022). The data from the study by Fujii et al., which included the clinicopathologic characteristics of 158 UTUC patients from their study, were downloaded(Fujii et al. 2021). Using the study by Fujii et al., survival analysis was also performed.

### Protein-protein interaction network analysis

The Search Tool for the Retrieval of Interacting Genes (STRING) (https://string-db.org/) and the Multiple Association Network Integration Algorithm (GeneMANIA) (http://www.genemania.org/) were used to construct the protein-protein interaction network between CSF3 and related proteins. In both databases, the species was set to *Homo sapiens*. In STRING, we set the minimum required interaction score to high confidence values (0.900) and limited the maximum number of interactors to 10. In GeneMANIA, we selected physical interactions, co-expression, predicted interactions, co-localization, genetic interactions, pathways, and shared protein domains to be included in the results.

### Statistical analysis

All statistical analyses were performed using SPSS (SPSS Inc., Chicago, IL, USA). Correlations between clinicopathological parameters and G-CSF expression were analyzed using Fisher’s exact test. Kaplan-Meier survival curves were constructed for G-CSF-positive and G-CSF-negative patients, and the survival rates of the two groups were compared. Differences between survival curves were tested for statistical significance by a log-rank test. Univariate and multivariate Cox proportional hazards regression analyses were performed to evaluate the associations between clinical covariates or various molecules and survival. G-CSF expression was compared between the two groups by the Wilcoxon signed-rank test. *P* values of < 0.05 were considered to indicate statistical significance. A receiver operating characteristic (ROC) curve analysis was performed, and the optimal cutoff value for G-CSF expression in TCs and SCs was determined using Youden’s index.

ROC curve, Concordance index (C-index) curve, Uniform Manifold Approximation and Projection (UMAP), and support vector machine (SVM) were also conducted by installing Python 3.0 on Jupyter Notebook (version 6.3.0), an interactive computing notebook environment. The codes used for these analyses are presented in Supplementary Data 1.

## Results

### Expression and distribution of G-CSF in UTUC tissues

**Fig. 1:**
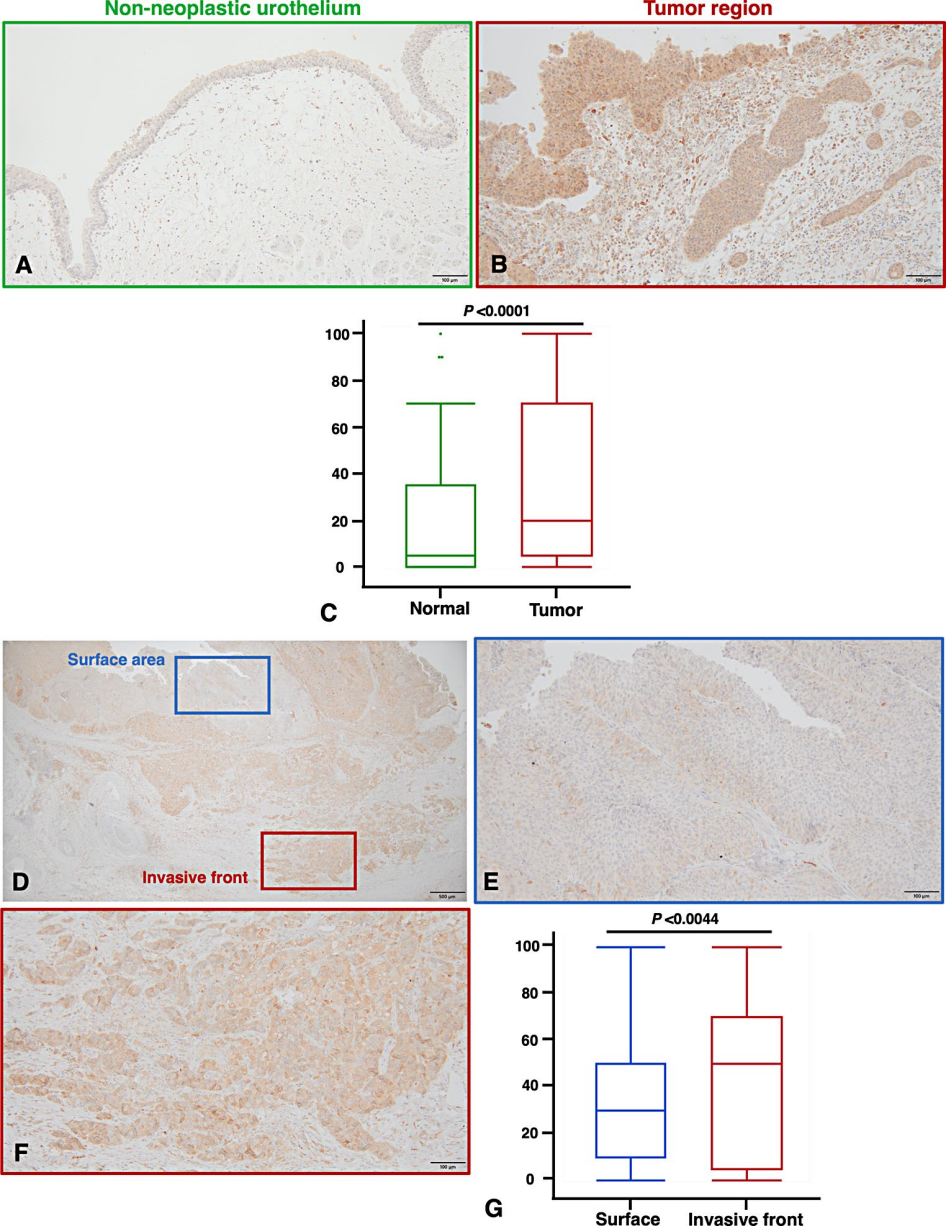
Immunohistochemical analysis of G-CSF expression in upper tract urothelial carcinoma (UTUC). (**A**) Representative staining image of G-CSF in adjacent non-neoplastic tissue. (**B**) Representative staining image of G-CSF in neoplastic tissue. (**A**, **B**) Scale bar indicates 100 μm. (**C**) Comparison of G-CSF expression levels between non-neoplastic and neoplastic lesions in UTUC tissues. Statistical significance was determined by the Wilcoxon signed-rank test. (**D**) Low-power magnification of G-CSF expression in UC tissues, showing both the surface area and the invasive front. Scale bar indicates 500 μm. (**E**) G-CSF expression on the tumor surface. (**F**) G-CSF expression at the tumor invasive front. (**E**, **F**) Scale bars indicate 100 μm. (**G**) Comparison of G-CSF expression levels between the surface area and the invasive front in UTUC tissues. Statistical significance was determined by the Wilcoxon signed-rank test. Scale bar indicates 100 μm

We first performed immunohistochemical staining of the 112 UTUC tissue samples. In non-neoplastic urothelium, the staining of G-CSF was weak or absent (Fig. 1A), whereas strong cytoplasmic expression of G-CSF was detected in UTUC tissues (Fig. 1B). Depending on the case, G-CSF expression was observed in both TCs and tumor SCs (Fig. 1B). In comparison to non-neoplastic urothelium, the expression levels of G-CSF were significantly higher in tumor tissues (*P* < 0.0001, Fig. 1C). Moreover, the cells expressing G-CSF were frequently observed in the deeper invasive region compared to the surface of the tumor (Fig. 1D–F). We also examined G-CSF expression in the surface area and the deeply invasive front. The expression of G-CSF was significantly higher in the invasive front than on the surface (*P* = 0.0044, Fig. 1G).

### Expression of G-CSF in UTUC and its relationship with clinicopathological parameters

We evaluated G-CSF expression in TCs using the H-score. The H-score of G-CSF was calculated with the following formula: H-score = 0 × % of non-stained cells + 1 × % of weakly stained cells + 2 × % of moderately stained cells + 3 × % of strongly stained cells (Fig. S1A). We then generated ROC curves to determine the cutoff value that correlated with CSS (Fig. S1B). With an optimal cutoff value of 50, specimens were classified as either negative (H-score < 50) or positive (H-score ≥ 50) for G-CSF expression in TCs. G-CSF-positive TCs were observed in 55 (49%) of the 112 cases of UTUC. They were associated with nodular/flat morphology (*P* < 0.0001), high tumor grade (*P* = 0.0237), advanced pathological T stage (*P* < 0.0001), concomitant CIS (*P* = 0.0327), and lympho-vascular invasion (*P* = 0.0331) (Table 1).

**Table 1:** Relationship between immunohistochemical positivity for GCSF in tumor cells and clinicopathological parameters in 112 cases of upper tract urothelial carcinoma

	GCSF expression in TCs			GCSF expression in SCs		
	Positive	Negative	*P* value	Positive	Negative	*P* value
Age						
** Average ± SD**	69.8 ± 11.0	73.7 ± 8.6	0.0592	70.7 ± 10.4	75.2 ± 7.9	**0.0346**
Sex						
Female (*n* = 30)	14 (47%)	16	0.8324	7 (23%)	23	1.0000
Man (*n* = 82)	41 (50%)	41		19 (23%)	63	
Location						
Renal pelvis (*n* = 56)	22 (39%)	34	0.1150	10 (18%)	46	0.3625
Ureter (*n* = 51)	30 (59%)	21		15 (29%)	36	
Both (*n* = 5)	3 (60%)	2		1 (20%)	4	
Morphology						
Papillary (*n* = 66)	21 (32%)	45	**< 0.0001**	7 (11%)	59	**0.0002**
** Nodular/Flat** (*n* = **46)**	34 (74%)	12		19 (41%)	27	
Histological classification						
Pure type (*n* = 103)	49 (48%)	54	0.3167	22 (21%)	81	0.2086
Variants (*n* = 9)	6 (67%)	3		4 (44%)	5	
Histological grade						
Low grade (*n* = 49)	18 (37%)	31	**0.0237**	6 (12%)	43	**0.0230**
** High grade** (*n* = **63)**	37 (59%)	26		20 (32%)	43	
Pathological T stage						
pTa/is/1 (*n* = 54)	15 (28%)	39	**< 0.0001**	2 (4%)	52	**< 0.0001**
** pT2/3/4** (*n* = **58)**	40 (69%)	18		24 (41%)	34	
Concomitant CIS						
Absence (*n* = 60)	24 (40%)	36	**0.0327**	9 (15%)	51	**0.0478**
** Presence** (*n* = **47)**	29 (62%)	18		17 (36%)	30	
Lympho-vascular invasion						
Ly 0 (*n* = 79)	34 (43%)	45	**0.0331**	14 (18%)	65	**0.0439**
** Ly 1** (*n* = **30)**	20 (67%)	10		11 (37%)	19	
Venous invasion						
v0 (*n* = 34)	21 (62%)	13	0.1008	14 (19%)	61	0.1420
v1 (*n* = 75)	33 (44%)	42		11 (32%)	23	

Bold values indicate statistical significance (*P* < 0.05)

*Abbreviations* G-CSF, Granulocyte-colony stimulating factor; TCs, tumor cells; SCs, stromal cells; SD, standard deviation; CIS, carcinoma in situ

*P* values were calculated with Fisher’s exact test

We also evaluated G-CSF expression in SCs. The percentages of G-CSF-expressing cells including fibroblasts, macrophages, and inflammatory cells were calculated in the tumor stromal area (Fig. S2A). After conducting ROC curve analysis, an optimal cutoff value of 70 was determined, and specimens were classified as either negative (< 70) or positive (≥ 70) for G-CSF expression in SCs (Fig. S2B). G-CSF-positive SCs were observed in 26 (23%) of the 112 cases of UTUC. They were associated with older age (0.0346), nodular/flat morphology (*P* = 0.0002), high tumor grade (*P* = 0.0230), advanced pathological T stage (*P* < 0.0001), concomitant CIS (*P* = 0.0478) and lympho-vascular invasion (*P* = 0.0439) (Table 1). G-CSF expression was not significantly associated with blood test results in either TCs or SCs (Table S1).

### Relationship between expression and prognosis of UTUC

**Fig. 2:**
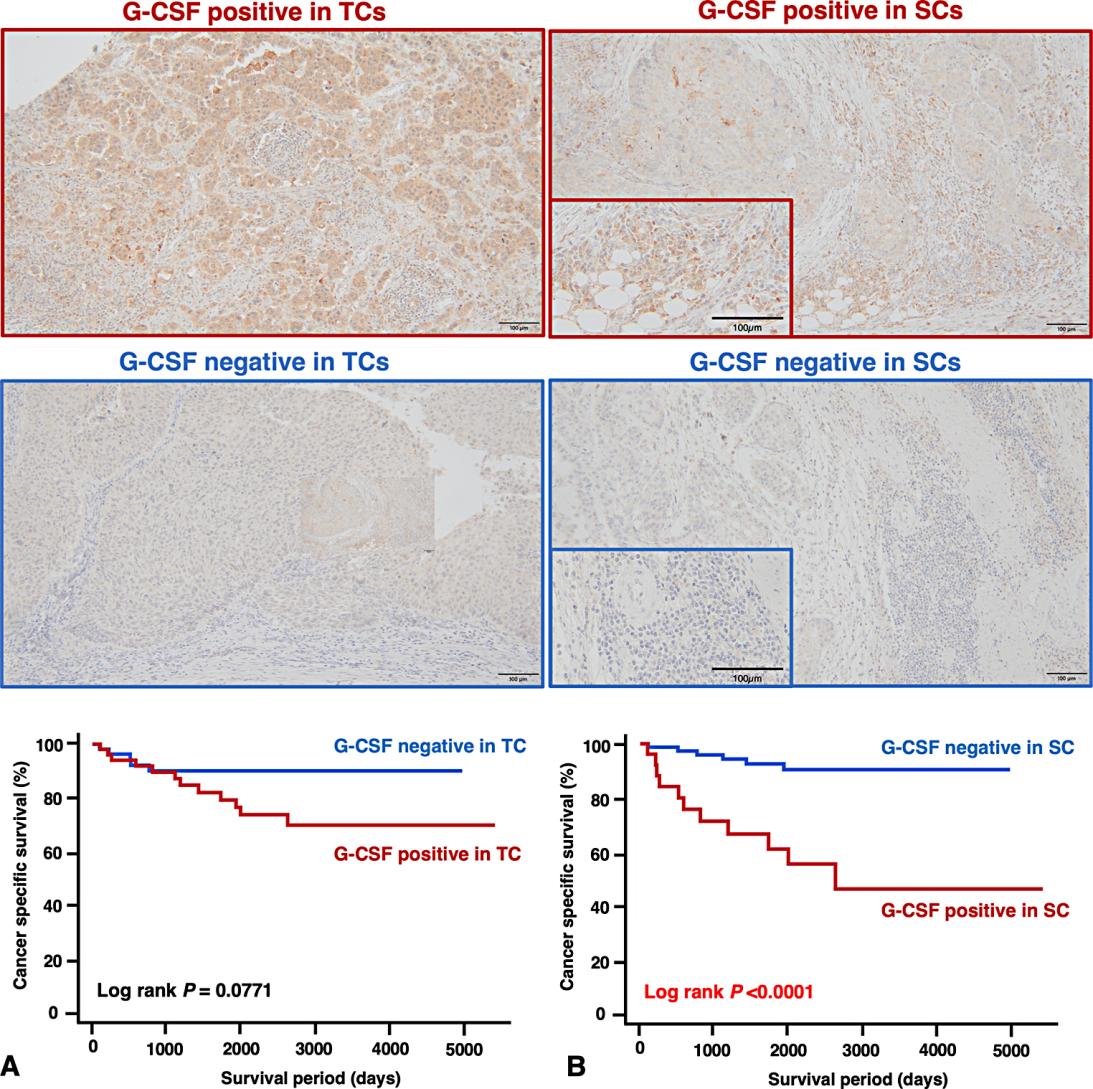
Survival analysis. (**A**) Representative staining images showing G-CSF positive and negative expression in tumor cells (TCs) along with a Kaplan-Meier plot for cancer-specific survival (CSS). (**B**) Representative staining images showing G-CSF positive and negative expression in stromal cells (SCs) along with a Kaplan-Meier plot for CSS. Scale bars indicate 100 μm

**Fig. 3:**
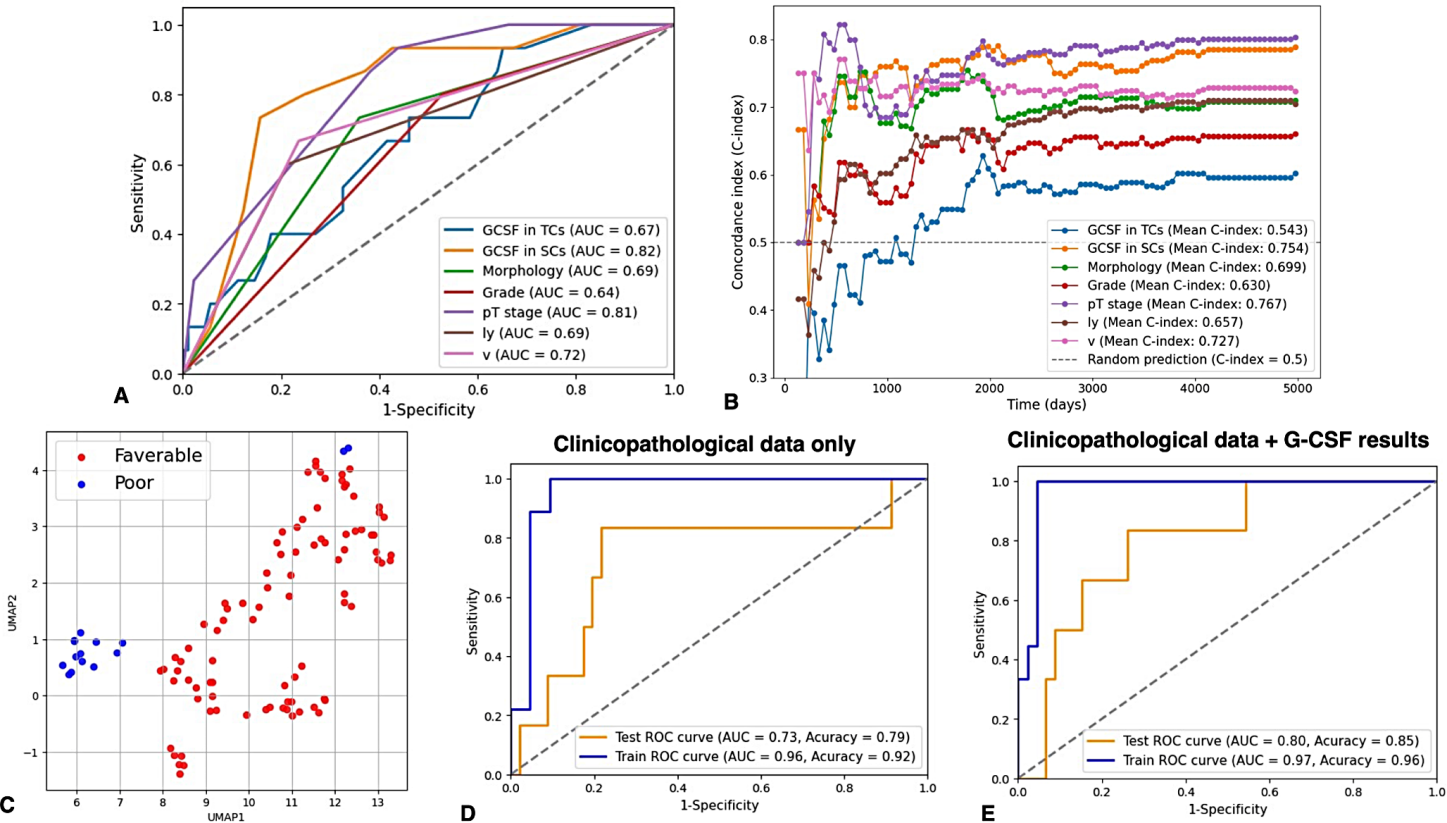
Survival analysis using a machine learning approach. (**A**) Receiver operating characteristic (ROC) curve of G-CSF status and other clinicopathological parameters. (**B**) The C-index curve of G-CSF status and other clinicopathological parameters. (**C**) Distribution plots for each upper tract urothelial carcinoma (UTUC) case generated using Uniform Manifold Approximation and Projection (UMAP). (**D**) ROC curves comparing the classification of prognosis using the clinicopathological dataset alone versus the combined clinicopathological dataset and G-CSF results (**E**) utilizing a support vector machine (SVM) algorithm

We performed a Kaplan-Meier analysis to investigate the association between G-CSF expression and patient prognosis to further elucidate the clinical impact of G-CSF on UTUC in our 112 patients. G-CSF expression in TCs was marginally significantly associated with increased CSS (*P* = 0.0771, Fig. 2A). G-CSF expression in SCs was significantly associated with increased CSS (*P* < 0.0001, Fig. 2B). We also performed univariate and multivariate Cox proportional hazards analyses. In the univariate analysis, morphology, tumor grade, T stage, lympho-vascular invasion, venous invasion, and G-CSF expression in SCs were associated with CSS (Table 2). In the multivariate models, G-CSF expression in SCs was the only independent prognostic marker for CSS (Table 2). In addition, G-CSF expression in SCs showed a stronger prognostic value compared to the key clinicopathological factors, as verified by the ROC and C-index analysis (Fig. 3A, B).

**Table 2:** Univariate and multivariate Cox proportional hazards analyses of cancer-specific survival in 112 cases of upper tract urothelial carcinoma

	Prediction of cancer specific survival			
	Univariate analysis		Multivariate analysis	
	HR (95% CI)	*P* value	OR (95% CI)	*P* value
Morphology				
Papillary	1 (Reference)	**0.0010**	1 (Reference)	0.2417
Nodular/Flat	6.45 (2.12–19.69)		1.85 (0.40–9.33)	
Histological classification				
Pure type	1 (Reference)	0.0630	1 (Reference)	0.4283
Variants	3.26 (0.94–11.35)		0.38 (0.02–2.89)	
Grade				
Low grade	1 (Reference)	**0.0323**	1 (Reference)	0.9091
High grade	3.37 (1.11–10.27)		1.1 (0.24–6.98)	
T stage				
Ta/is/1	1 (Reference)	**< 0.0001**	1 (Reference)	0.1749
T2/3/4	20.71 (2.75-155.79)		6.11 (0.59-149.86)	
Concomitant CIS				
Absence	1 (Reference)	0.2458	1 (Reference)	0.2830
Presence	1.76 (0.68–4.57)		0.55(0.18–1.70)	
Lympho-vascular invasion				
Ly 0	1 (Reference)	**0.0003**	1 (Reference)	0.2657
Ly 1	5.88 (2.26–15.28)		2.13 (0.59–8.85)	
Venous invasion				
v0	1 (Reference)	**0.0037**	1 (Reference)	0.2758
v1	4.08 (1.58–10.55)		2.11 (0.57–8.65)	
GCSF in TCs				
Positive	1 (Reference)	0.0876	1 (Reference)	0.2417
Negative	2.48 (0.87–7.05)		0.40 (0.08–1.86)	
GCSF in SCs				
Positive	1 (Reference)	**0.0001**	1 (Reference)	**0.0198**
Negative	6.87 (2.61–19.97)		6.42 (1.54–36.55)	

Bold values indicate statistical significance (*P* < 0.05)

*Abbreviations* HR, hazard ratio; CI, confidence interval; G-CSF, granulocyte-colony stimulating factor; TCs, tumor cells; SCs, stromal cells; CIS, carcinoma in situ

Using all data from a clinicopathological dataset (shown in Table 1), including G-CSF expression in both TCs and SCs, UMAP was applied to visualize distribution plots. We observed a tendency to distinguish between favorable and poor prognosis through the reduction of dimensionality (Fig. 3C). We then constructed a prognostic model using a SVM to predict CSS. The dataset was split into training and testing sets using a 50/50 ratio to evaluate the classification model. The prognostic risk model based solely on the clinicopathological dataset achieved AUC of 0.73 and 0.96, accuracy of 0.79 and 0.92 for the training and testing models, respectively (Fig. 3D). Interestingly, the predictive performance improved by incorporating G-CSF expression results into the clinicopathological dataset (testing/training AUC and accuracy: 0.80/0.97, 0.85/0.96, respectively) (Fig. 3E).

### In silico analysis of G-CSF expression

We then evaluated the relationship between G-CSF expression and several parameters using The Cancer Genome Atlas UTUC dataset. G-CSF expression was significantly higher in UTUC tissues compared to normal urothelium in the GSE166912 study (Fig. S3A). Increased G-CSF expression was observed in cases with higher T stage, higher histological grade, the presence of sarcomatoid changes, and the TP53 mutational subtype in the study by Fujii et al. [21]. (Fig. S3B-E). Notably, high G-CSF expression was significantly associated with poor progression-free survival (*P* = 0.003, Fig. S3F) and disease-specific survival (*P* < 0.001, Fig. S3G) among UTUC patients in the same study.

### Correlation between G-CSF expression and various cancer‑related molecules

Although we showed that G-CSF could contribute to tumor progression in UTUC, the molecules with which G-CSF is associated remain largely unknown. We therefore investigated the relationship between G-CSF expression and various cancer-related molecules, including Ki-67, PD-L1, CD8, CD44v9, HER2, EGFR, FGFR3, p53, GATA3, and CK5/6 in the 112 cases of UTUC. We revealed that G-CSF expression in both TCs and SCs was significantly associated with the high expression of Ki-67, PD-L1, HER2, and p53 (Table 3). We also investigated whether G-CSF has an independent predictive value for prognosis compared to other markers. We performed univariate and multivariate Cox proportional hazards analyses of CSS for various molecules including those with G-CSF expression. In the univariate analysis, G-CSF (SCs), PD-L1 (TCs and TILs), and CD44v9 were associated with CSS. Among them, G-CSF expression in SCs had the highest hazard ratio. In the multivariate models, the expression of G-CSF (SCs), CD44v9, p53, and CK 5/6 provided an independent prognostic marker for CSS (Table S2).

**Table 3:** Relationship between the expression of G-CSF and various cancer-related molecules in 112 cases of upper tract urothelial carcinoma

	GCSF expression in TCs			GCSF expression in SCs		
	Positive	Negative	*P* value	Positive	Negative	*P* value
Ki-67						
Positive (> 20%)	22 (76%)	7	**0.0011**	11 (38%)	18	**0.0407**
Negative (≤ 20%)	33 (40%)	50		15 (18%)	68	
PD-L1 in TCs						
Positive	11 (69%)	5	0.1096	8 (50%)	8	**0.0108**
Negative	44 (46%)	52		18 (19%)	78	
PD-L1 in TILs						
Positive	23 (68%)	11	**0.0133**	13 (38%)	21	**0.0165**
Negative	32 (41%)	46		13 (17%)	65	
CD8 in TCs						
Positive	19 (61%)	12	0.1403	7 (23%)	24	1.0000
Negative	36 (44%)	45		19 (23%)	62	
CD44v9						
Positive	14 (56%)	11	0.3705	6 (24%)	19	0.7808
Negative	38 (45%)	46		17 (20%)	67	
HER2						
Positive	16 (80%)	4	**0.0028**	11 (55%)	9	**0.0006**
Negative	39 (42%)	53		15 (16%)	77	
EGFR						
Positive	17 (61%)	11	0.1925	10 (36%)	18	0.1187
Negative	38 (45%)	46		16 (19%)	68	
FGFR3						
Positive	14 (39%)	22	0.1597	6 (17%)	30	0.3401
Negative	41 (54%)	35		20 (26%)	56	
p53						
Positive	23 (72%)	9	**0.0032**	15 (47%)	17	**0.0004**
Negative	32 (40%)	48		11 (14%)	69	
GATA3						
Positive	46 (46%)	54	0.0713	21 (21%)	79	0.1452
Negative	9 (75%)	3		5 (42%)	7	
CK 5/6						
Positive	14 (58%)	10	0.3612	4 (17%)	20	0.5859
Negative	41 (47%)	47		22 (25%)	66	

Bold values indicate statistical significance (*P* < 0.05)

*Abbreviations* G-CSF, granulocyte-colony stimulating factor; TCs, tumor cells; SCs, stromal cells; PD-L1, programmed death ligand 1; TILs, tumor infiltrating lymphocyte; CD44v9, CD44 variant 9; HER2, human epidermal growth factor receptor type 2; EGFR, epidermal growth factor receptor; FGFR3, fibroblast growth factor receptor; GATA3, GATA binding protein 3; CK 5/6, cytokeratin 5/6

*P* values were calculated with Fisher’s exact test

### Protein-protein interaction network of G-CSF

As G-CSF may interact with many proteins or genes to influence tumorigenesis, it is important to identify the proteins with which it interacts. The STRING database was used to create a protein-protein interaction network, which provides interaction evidence through text mining, experiments, databases, co-expression, neighborhood, gene fusion, and co-occurrence. The top 10 proteins interacting with G-CSF were CSF3R, THPO, EPO, JAK2, CSF2, IFNG, CCL11, IL1A, IL1B, and IL6 (Fig. S4A). We also confirmed these interactions using the GeneMANIA database, which constructs complex gene-gene functional interaction networks. Twenty genes associated with G-CSF were identified, including G-CSF3R, ELANE, IL6, IL6ST, POU2F2, HSF1, SFTPB, CXCL3, TNFAIP6, HBEGF, AGER, EDN1, RELA, CCL2, LIF, RARRES1, NPHP1, CAMLG, GTF3C1, and SLC34A2 (Fig. S4B). Therefore, G-CSF is suggested to be associated with immune responses, inflammatory cytokines, hematopoietic hormones, chemokines, proteases, and cancer-related molecules.

## Discussion

G-CSF has been shown to be involved in tumor growth, progression, and metastasis in many cancers (Karagiannidis et al. 2021). However, the clinicopathological significance of G-CSF expression in UTUC remains poorly understood. In the present study, our immunohistochemical analysis showed that G-CSF expression was significantly higher in tumor tissues than in normal urothelium and was associated with nodular/flat morphology, high tumor grade, advanced pathological T stage, concomitant CIS, and lympho-vascular invasion. Similar findings were confirmed through in silico analysis. Significant G-CSF expression was more frequently observed in the invasive front than in the superficial area. In addition to that in TCs, strong G-CSF expression was occasionally observed in tumor SCs. In fact, it has been reported that G-CSF is expressed not only in TCs but also in immune cells, fibroblasts, and macrophages (Karagiannidis et al. 2021; Cetean et al. 2015). Stromal G-CSF expression was also associated with adverse clinicopathological features and poor prognosis in UTUC. Interestingly, stromal G-CSF expression emerged as a more predictive biomarker for poor prognosis and was an independent prognostic factor among the significant clinicopathological factors in UTUC. In silico analysis showed high expression of G-CSF to be significantly associated with decreased progression-free survival and disease-specific survival. Moreover, constructing the SVM model that incorporated G-CSF expression alongside clinicopathological factors predicted poor prognosis with greater accuracy than using clinicopathological factors alone. These results suggest that G-CSF expression may play an important role in the TMEs of UTUC, with stromal G-CSF expression potentially contributing to poor prognosis.

G-CSF is reported to be associated with various signaling pathways(Dwivedi and Greis 2017; Furmento et al. 2014; Karagiannidis et al. 2021). According to the literature, G-CSF activates STAT through interaction with JAK2 to promote tumor cell proliferation in several cancers (Karagiannidis et al. 2021; Kumar et al. 2014; Fan et al. 2018; Li et al. 2016; Agarwal et al. 2015). Indeed, our findings showed that G-CSF expression correlated with Ki-67, indicating that G-CSF is involved in tumor proliferation in UTUC. STRING analysis confirmed a strong relationship between JAK2 and G-CSF, as well as with proinflammatory cytokines. Furthermore, our immunohistochemical analysis confirmed a significant correlation between G-CSF and HER2. It is well known that JAK2 activation is associated with HER2 positivity in breast cancer(Regua et al. 2024; Doheny et al. 2020). Thus, G-CSF may be linked to JAK2 and various cytokines and chemokines that, when influenced by stimulation of HER2 signaling, contribute to tumor progression, tumorigenesis, and poor prognosis in UTUC. G-CSF also formed a network with other proteins involved in cancer growth and progression, such as POU2F2, HEF1, and CXCL3. These results suggest that G-CSF may play a role in the progression of UTUC and could serve as a potential biomarker and therapeutic target to support personalized treatment strategies.

We also showed an association between G-CSF and PD-L1 expression in UTUC. The PD-1/PD-L1 pathway is one of the most important signaling pathways in immune checkpoint therapy (Han et al. 2020). The expression status of PD-L1 is known to be related to the efficacy of immunotherapy (Han et al. 2020). In UTUC, PD-L1 expression is associated with adverse pathological features and poor prognosis (Chen et al. 2021a, b; Lu et al. 2020). According to the network analysis, G-CSF closely interacted with IL-6 (interleukin 6), a pleiotropic cytokine. IL-6 is a promoter of G-CSF, is associated with tumor progression, and is thought to influence anti-tumor immunity through various mechanisms (Jones and Jenkins 2018; Karagiannidis et al. 2021; Liu et al. 2020). It is also known to induce PD-L1 expression via the JAK/STAT3 pathway (Chan et al. 2019; Rahmadiani et al. 2024; Jiang et al. 2019). Thus, it is possible that the interaction between G-CSF and IL-6 could influence PD-L1 expression, and G-CSF expression may have potential as a predictor of UTUC response to immunotherapy.

Fujii et al. showed in 2021 that UTUC can be divided into five DNA-based molecular subtypes (i.e., hypermutated, TP53/MDM2, RAS, FGFR3, and triple negative), and the TP53/MDM2 mutational subtype is associated with high grade, invasiveness, and a poor prognosis in UTUC (Fujii et al. 2021). Thus, G-CSF expression was similar to the TP53/MDM2 mutational subtype in clinicopathological features. Interestingly, in addition to G-CSF being significantly associated with p53 expression in our immunohistochemical analysis, G-CSF expression was significantly higher in the TP53 mutational subtype in our in silico analysis. Immunohistochemical positivity for p53 represents TP53 gene mutations. Therefore, there might be a close association between G-CSF and TP53/MDM2 mutational subtype in UTUC. Additionally, low‐grade papillary UC predominantly follows the FGFR3 signaling pathway, whereas nodular/flat UC follows the TP53 pathway(Inamura 2018; Pietzak et al. 2017). Taken together, G-CSF could be one of the important molecules in the carcinogenesis of UC by participating in TP53 pathway activation.

This study has some limitations. First, it was retrospective in nature and might have potential bias due to patient selection. Therefore, a prospective study is necessary. Second, although we showed the prognostic significance of stromal G-CSF expression in UTUC, the number of patients included was limited. Additionally, in UTUC cases with poor prognosis, it remains unclear which types of stromal cells, such as T and B lymphocytes, neutrophils, fibroblasts, or macrophages, express G-CSF. Further research with a larger study population is needed to determine the stromal distribution of G-CSF expression and to explore its detailed relationship with prognosis in UTUC. This could lead to the development of a more accurate prognostic model for predicting disease progression. Third, the detailed molecular mechanisms underlying the relationships between G-CSF expression, PD-L1, HER2, and p53 in UTUC remain unclear. Further studies are needed to elucidate these molecular activities in tumor cell biology.

## Conclusions

To the best of our knowledge, this is the first study to comprehensively characterize G-CSF expression in UTUC. We found that G-CSF was expressed in TCs and SCs and that its expression was associated with adverse clinicopathological features. Stromal G-CSF expression emerged as the more predictive prognostic factor. Additionally, G-CSF expression was associated with Ki-67, PD-L1, HER2, and p53. Identifying prognostic factors that accurately predict outcomes is crucial for determining the appropriate treatment for patients with UTUC. Taken together, evaluating G-CSF expression may provide insights that could support clinical decision-making.

## Electronic supplementary material

Below is the link to the electronic supplementary material.

Supplementary Material 1

Supplementary Material 2

Supplementary Material 3

Supplementary Material 4

Supplementary Material 5

Supplementary Material 6

## Data Availability

All research data and material will be made available upon request. Most data are included in the main manuscript.

## References

[CR1] Abouassaly R, Alibhai SM, Shah N, Timilshina N, Fleshner N, Finelli A (2010) Troubling outcomes from population-level analysis of surgery for upper tract urothelial carcinoma. Urology 76(4):895–901. 10.1016/j.urology.2010.04.020 20646743 10.1016/j.urology.2010.04.020

[CR2] Agarwal S, Lakoma A, Chen Z, Hicks J, Metelitsa LS, Kim ES et al (2015) G-CSF promotes Neuroblastoma Tumorigenicity and Metastasis via STAT3-Dependent Cancer stem cell activation. Cancer Res 75(12):2566–2579. 10.1158/0008-5472.CAN-14-2946 25908586 10.1158/0008-5472.CAN-14-2946 PMC4470771

[CR3] Cetean S, Căinap C, Constantin AM, Căinap S, Gherman A, Oprean L et al (2015) The importance of the granulocyte-colony stimulating factor in oncology. Clujul Med 88(4):468–472. 10.15386/cjmed-531 26732055 10.15386/cjmed-531 PMC4689238

[CR4] Chan LC, Li CW, Xia W, Hsu JM, Lee HH, Cha JH et al (2019) IL-6/JAK1 pathway drives PD-L1 Y112 phosphorylation to promote cancer immune evasion. J Clin Invest 129(8):3324–3338. 10.1172/JCI126022 31305264 10.1172/JCI126022 PMC6668668

[CR5] Chen CH, Tsai MY, Chiang PC, Sung MT, Luo HL, Suen JL et al (2021a) Prognostic value of PD-L1 combined positive score in patients with upper tract urothelial carcinoma. Cancer Immunol Immunother 70(10):2981–2990. 10.1007/s00262-021-02890-y 33740124 10.1007/s00262-021-02890-y PMC10992487

[CR6] Chen J, Zhong W, Yang M, Hou W, Wang X, Xia K et al (2021b) Development and validation of a PD-L1/PD-1/CD8 axis-based classifier to predict cancer survival of upper tract urothelial carcinoma after radical nephroureterectomy. Cancer Immunol Immunother 70(9):2657–2668. 10.1007/s00262-020-02827-x 33606065 10.1007/s00262-020-02827-x PMC10992229

[CR7] Doheny D, Sirkisoon S, Carpenter RL, Aguayo NR, Regua AT, Anguelov M et al (2020) Combined inhibition of JAK2-STAT3 and SMO-GLI1/tGLI1 pathways suppresses breast cancer stem cells, tumor growth, and metastasis. Oncogene 39(42):6589–6605. 10.1038/s41388-020-01454-1 32929154 10.1038/s41388-020-01454-1 PMC7572897

[CR8] Dwivedi P, Greis KD (2017) Granulocyte colony-stimulating factor receptor signaling in severe congenital neutropenia, chronic neutrophilic leukemia, and related malignancies. Exp Hematol 46:9–20. 10.1016/j.exphem.2016.10.008 27789332 10.1016/j.exphem.2016.10.008 PMC5241233

[CR9] Fan Z, Li Y, Zhao Q, Fan L, Tan B, Zuo J et al (2018) Highly expressed Granulocyte colony-stimulating factor (G-CSF) and granulocyte colony-stimulating factor receptor (G-CSFR) in human gastric Cancer leads to poor survival. Med Sci Monit 24:1701–1711. 10.12659/msm.909128 29567938 10.12659/MSM.909128 PMC5880331

[CR10] Favaretto RL, Zequi SC, Oliveira RAR, Santana T, Costa WH, Cunha IW et al (2018) Tissue-based molecular markers in upper tract urothelial carcinoma and their prognostic implications. Int Braz J Urol 44(1):22–37. 10.1590/S1677-5538.IBJU.2017.0204 29135410 10.1590/S1677-5538.IBJU.2017.0204 PMC5815529

[CR11] Fujii Y, Sato Y, Suzuki H, Kakiuchi N, Yoshizato T, Lenis AT et al (2021) Molecular classification and diagnostics of upper urinary tract urothelial carcinoma. Cancer Cell 39(6):793–809e8. 10.1016/j.ccell.2021.05.008 34129823 10.1016/j.ccell.2021.05.008 PMC9110171

[CR12] Furmento VA, Marino J, Blank VC, Roguin LP (2014) The granulocyte colony-stimulating factor (G-CSF) upregulates metalloproteinase-2 and VEGF through PI3K/Akt and Erk1/2 activation in human trophoblast swan 71 cells. Placenta 35(11):937–946. 10.1016/j.placenta.2014.09.003 25249155 10.1016/j.placenta.2014.09.003

[CR13] Hagiwara M, Kikuchi E, Kosaka T, Mikami S, Saya H, Oya M (2016) Variant isoforms of CD44 expression in upper tract urothelial cancer as a predictive marker for recurrence and mortality. Urol Oncol 34(8):337e19–337e26. 10.1016/j.urolonc.2016.03.015 10.1016/j.urolonc.2016.03.015 27133224

[CR14] Han Y, Liu D, Li L (2020) PD-1/PD-L1 pathway: current researches in cancer. Am J Cancer Res 10(3):727–742 32266087 PMC7136921

[CR15] He K, Liu X, Hoffman RD, Shi RZ, Lv GY, Gao JL (2022) G-CSF/GM-CSF-induced hematopoietic dysregulation in the progression of solid tumors. FEBS Open Bio 12(7):1268–1285. 10.1002/2211-5463.13445 35612789 10.1002/2211-5463.13445 PMC9249339

[CR16] Inamura K (2018) Bladder Cancer: New insights into its Molecular Pathology. Cancers (Basel) 10(4). 10.3390/cancers10040100 10.3390/cancers10040100 PMC5923355 29614760

[CR17] Jiang X, Wang J, Deng X, Xiong F, Ge J, Xiang B et al (2019) Role of the tumor microenvironment in PD-L1/PD-1-mediated tumor immune escape. Mol Cancer 18(1):10. 10.1186/s12943-018-0928-4 30646912 10.1186/s12943-018-0928-4 PMC6332843

[CR18] Jones SA, Jenkins BJ (2018) Recent insights into targeting the IL-6 cytokine family in inflammatory diseases and cancer. Nat Rev Immunol 18(12):773–789. 10.1038/s41577-018-0066-7 30254251 10.1038/s41577-018-0066-7

[CR19] Karagiannidis I, Salataj E, Said Abu Egal E, Beswick EJ (2021) G-CSF in tumors: aggressiveness, tumor microenvironment and immune cell regulation. Cytokine 142:155479. 10.1016/j.cyto.2021.155479 33677228 10.1016/j.cyto.2021.155479 PMC8044051

[CR20] Karanović S, Ardin M, Tang Z, Tomić K, Villar S, Renard C et al (2022) Molecular profiles and urinary biomarkers of upper tract urothelial carcinomas associated with aristolochic acid exposure. Int J Cancer 150(2):374–386. 10.1002/ijc.33827 34569060 10.1002/ijc.33827 PMC8627473

[CR21] Kobayashi G, Hayashi T, Sentani K, Babasaki T, Sekino Y, Inoue S et al (2021) Clinicopathological significance of claspin overexpression and its efficacy as a novel biomarker for the diagnosis of urothelial carcinoma. Virchows Arch. 10.1007/s00428-021-03239-7 34842980 10.1007/s00428-021-03239-7

[CR22] Kobayashi G, Hayashi T, Sentani K, Ikeda K, Babasaki T, Shigematsu Y et al (2022a) ANXA10 Expression Is Inversely Associated with Tumor Stage, Grade, and TP53 Expression in Upper and Lower Urothelial Carcinoma. *Pathobiology*, 1–10. 10.1159/000524989 10.1159/000524989 35780773

[CR23] Kobayashi G, Hayashi T, Sentani K, Takemoto K, Sekino Y, Uraoka N et al (2022b) Clinicopathological significance of the overexpression of MUC1 in upper tract urothelial carcinoma and possible application as a diagnostic marker. Pathol Int. 10.1111/pin.13274 36169278 10.1111/pin.13274

[CR24] Kobayashi G, Hayashi T, Sentani K, Uraoka N, Fukui T, Kido A et al (2023a) MCM4 expression is associated with high-grade histology, tumor progression and poor prognosis in urothelial carcinoma. Diagn Pathol 18(1):106. 10.1186/s13000-023-01392-y 37737200 10.1186/s13000-023-01392-y PMC10515259

[CR25] Kobayashi G, Hayashi T, Sentani K, Uraoka N, Fukui T, Kido A et al (2023b) Clinicopathological significance of TUBB3 in upper tract urothelial carcinoma and possible application in urine cytology. Pathol Int 73(9):444–455. 10.1111/pin.13362 37589430 10.1111/pin.13362

[CR26] Kolawa A, D’Souza A, Tulpule V (2023) Overview, diagnosis, and Perioperative systemic therapy of Upper Tract Urothelial Carcinoma. Cancers (Basel) 15(19). 10.3390/cancers15194813 10.3390/cancers15194813 PMC10571968 37835507

[CR27] Kumar J, Fraser FW, Riley C, Ahmed N, McCulloch DR, Ward AC (2014) Granulocyte colony-stimulating factor receptor signalling via Janus kinase 2/signal transducer and activator of transcription 3 in ovarian cancer. Br J Cancer 110(1):133–145. 10.1038/bjc.2013.673 24220695 10.1038/bjc.2013.673 PMC3887286

[CR28] Leow JJ, Chong KT, Chang SL, Bellmunt J (2016) Upper tract urothelial carcinoma: a different disease entity in terms of management. ESMO Open 1(6):e000126. 10.1136/esmoopen-2016-000126 28848663 10.1136/esmoopen-2016-000126 PMC5419214

[CR29] Li W, Zhang X, Chen Y, Xie Y, Liu J, Feng Q et al (2016) G-CSF is a key modulator of MDSC and could be a potential therapeutic target in colitis-associated colorectal cancers. Protein Cell 7(2):130–140. 10.1007/s13238-015-0237-2 26797765 10.1007/s13238-015-0237-2 PMC4742385

[CR30] Li X, Cui M, Gu X, Fang D, Li H, Qin S et al (2020) Pattern and risk factors of local recurrence after nephroureterectomy for upper tract urothelial carcinoma. World J Surg Oncol 18(1):114. 10.1186/s12957-020-01877-w 32473636 10.1186/s12957-020-01877-w PMC7261378

[CR31] Liu L, Liu Y, Yan X, Zhou C, Xiong X (2020) The role of granulocyte colony–stimulating factor in breast cancer development: a review. Mol Med Rep 21(5):2019–2029. 10.3892/mmr.2020.11017 32186767 10.3892/mmr.2020.11017 PMC7115204

[CR32] Lu Y, Kang J, Luo Z, Song Y, Tian J, Li Z et al (2020) The prevalence and prognostic role of PD-L1 in Upper Tract Urothelial Carcinoma patients underwent Radical Nephroureterectomy: a systematic review and Meta-analysis. Front Oncol 10:1400. 10.3389/fonc.2020.01400 32974145 10.3389/fonc.2020.01400 PMC7472102

[CR33] Margulis V, Shariat SF, Matin SF, Kamat AM, Zigeuner R, Kikuchi E et al (2009) Outcomes of radical nephroureterectomy: a series from the Upper Tract Urothelial Carcinoma collaboration. Cancer 115(6):1224–1233. 10.1002/cncr.24135 19156917 10.1002/cncr.24135

[CR34] Pietzak EJ, Bagrodia A, Cha EK, Drill EN, Iyer G, Isharwal S et al (2017) Next-generation sequencing of nonmuscle invasive bladder Cancer reveals potential biomarkers and rational therapeutic targets. Eur Urol 72(6):952–959. 10.1016/j.eururo.2017.05.032 28583311 10.1016/j.eururo.2017.05.032 PMC6007852

[CR35] Rahmadiani N, Norahmawati E, Endharti AT, Hambalie AO, Isma SPP (2024) PD-L1, STAT3, IL6, and EGFR immunoexpressions in High-Grade Osteosarcoma. Adv Orthop 2024:9036225. 10.1155/2024/9036225 38434518 10.1155/2024/9036225 PMC10907101

[CR36] Regua AT, Bindal S, Najjar MK, Zhuang C, Khan M, Arrigo ABJ et al (2024) Dual inhibition of the TrkA and JAK2 pathways using entrectinib and pacritinib suppresses the growth and metastasis of HER2-positive and triple-negative breast cancers. Cancer Lett 597:217023. 10.1016/j.canlet.2024.217023 38852701 10.1016/j.canlet.2024.217023 PMC11533721

[CR37] Rouprêt M, Babjuk M, Compérat E, Zigeuner R, Sylvester RJ, Burger M et al (2018) European Association of Urology Guidelines on Upper urinary tract Urothelial Carcinoma: 2017 update. Eur Urol 73(1):111–122. 10.1016/j.eururo.2017.07.036 28867446 10.1016/j.eururo.2017.07.036

[CR38] Wang Q, Shao X, Zhang Y, Zhu M, Wang FXC, Mu J et al (2023) Role of tumor microenvironment in cancer progression and therapeutic strategy. Cancer Med 12(10):11149–11165. 10.1002/cam4.5698 36807772 10.1002/cam4.5698 PMC10242329

[CR39] Yan M, Zheng M, Niu R, Yang X, Tian S, Fan L et al (2022) Roles of tumor-associated neutrophils in tumor metastasis and its clinical applications. Front Cell Dev Biol 10:938289. 10.3389/fcell.2022.938289 36060811 10.3389/fcell.2022.938289 PMC9428510

