# Supplementary figures and images for: Distribution and clinicopathological characteristics of G-CSF expression in tumor cells and stromal cells in upper tract urothelial carcinoma
Source: J Cancer Res Clin Oncol. 2024 Dec 30;151(1):18. doi: 10.1007/s00432-024-06045-1 (PMC11685250; doi:10.1007/s00432-024-06045-1)

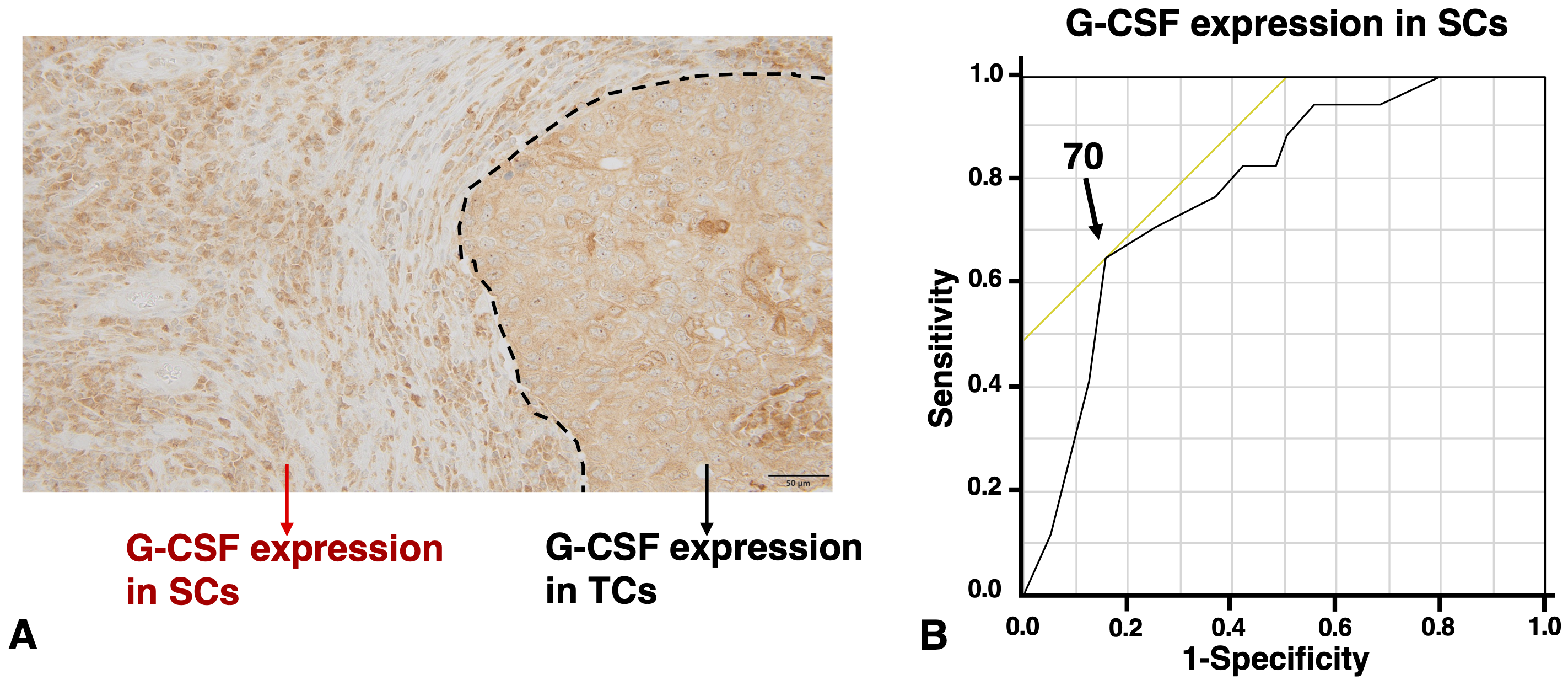

Supplement: Supplementary file 1 — Supplementary Material 1 [file 432_2024_6045_MOESM1_ESM.tiff]

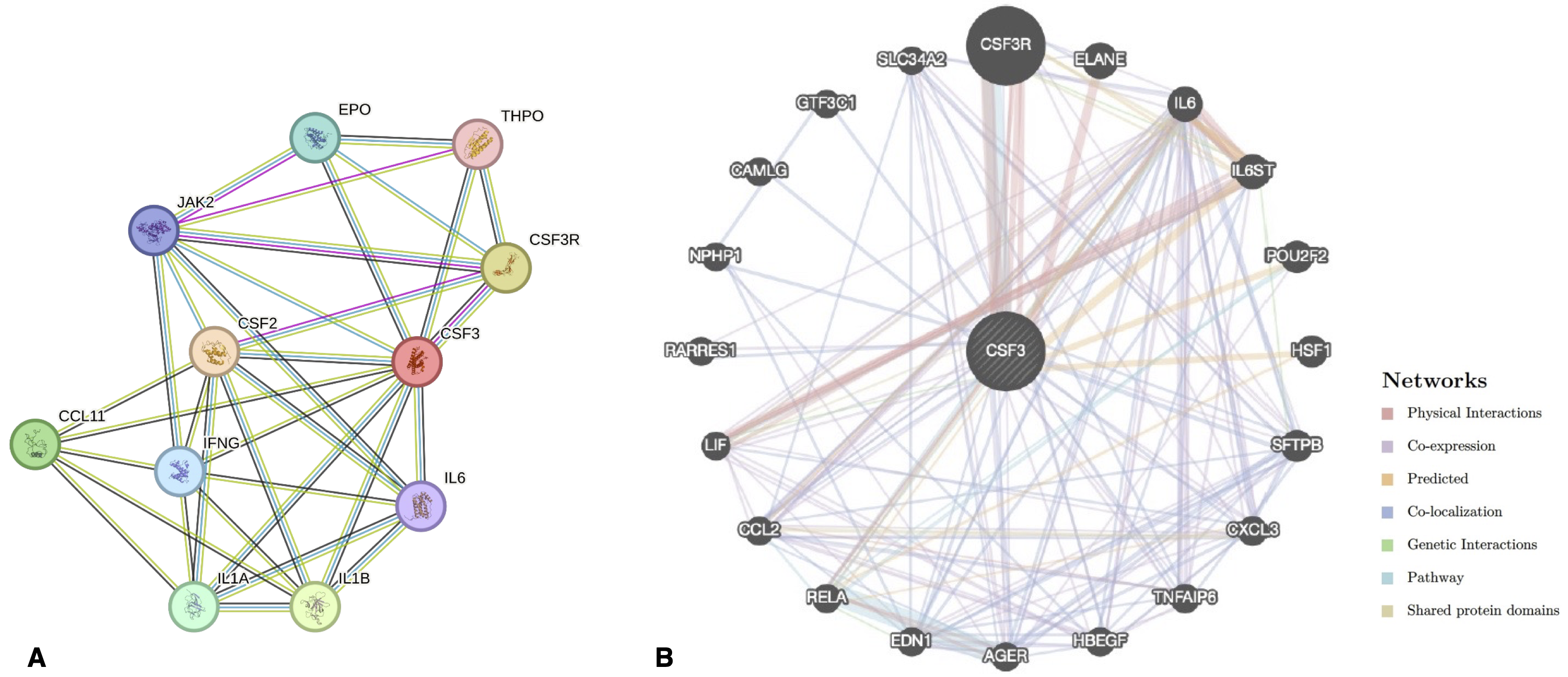

Supplement: Supplementary file 2 — Supplementary Material 2 [file 432_2024_6045_MOESM2_ESM.tiff]

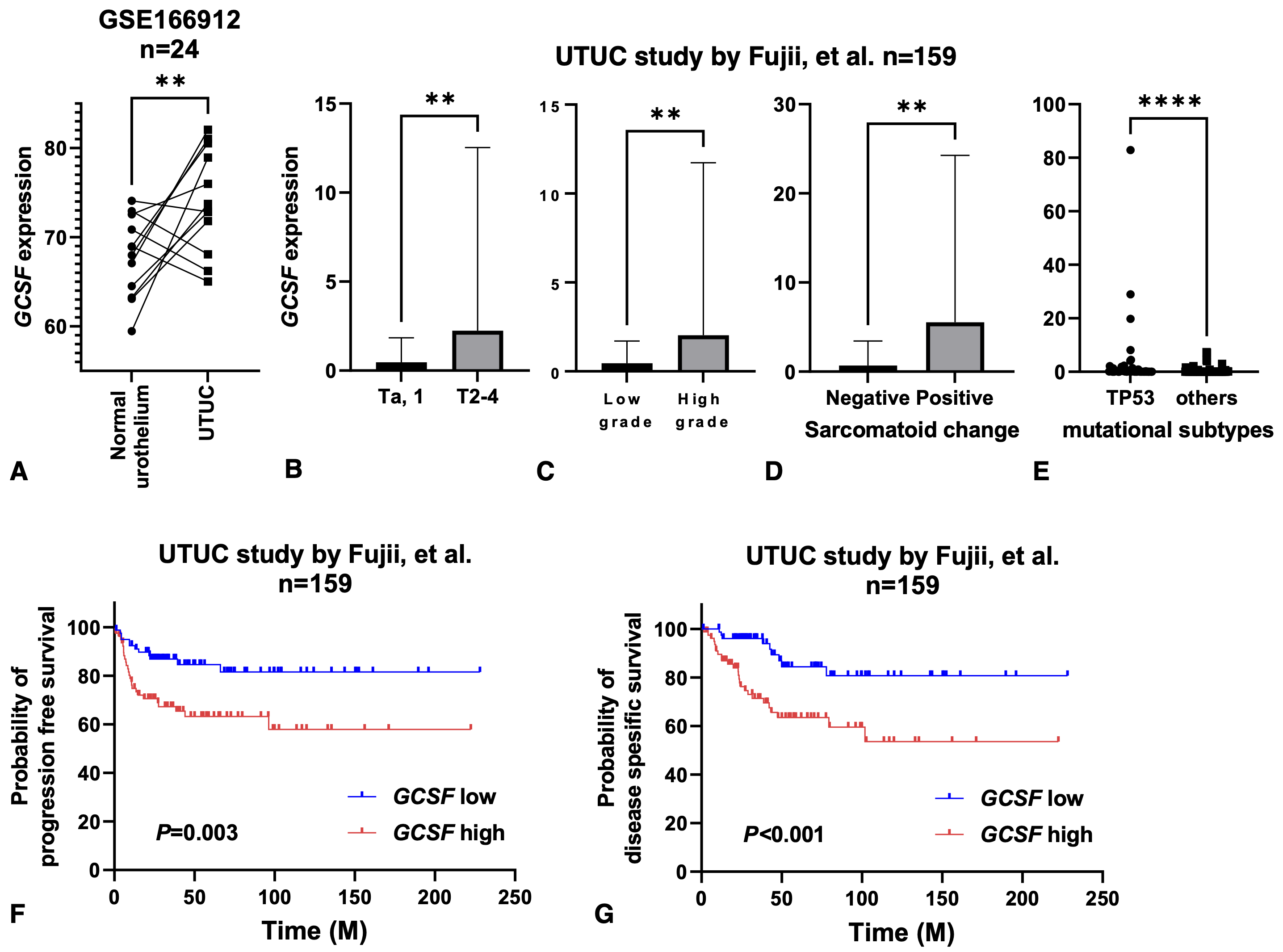

Supplement: Supplementary file 3 — Supplementary Material 3 [file 432_2024_6045_MOESM3_ESM.tiff]

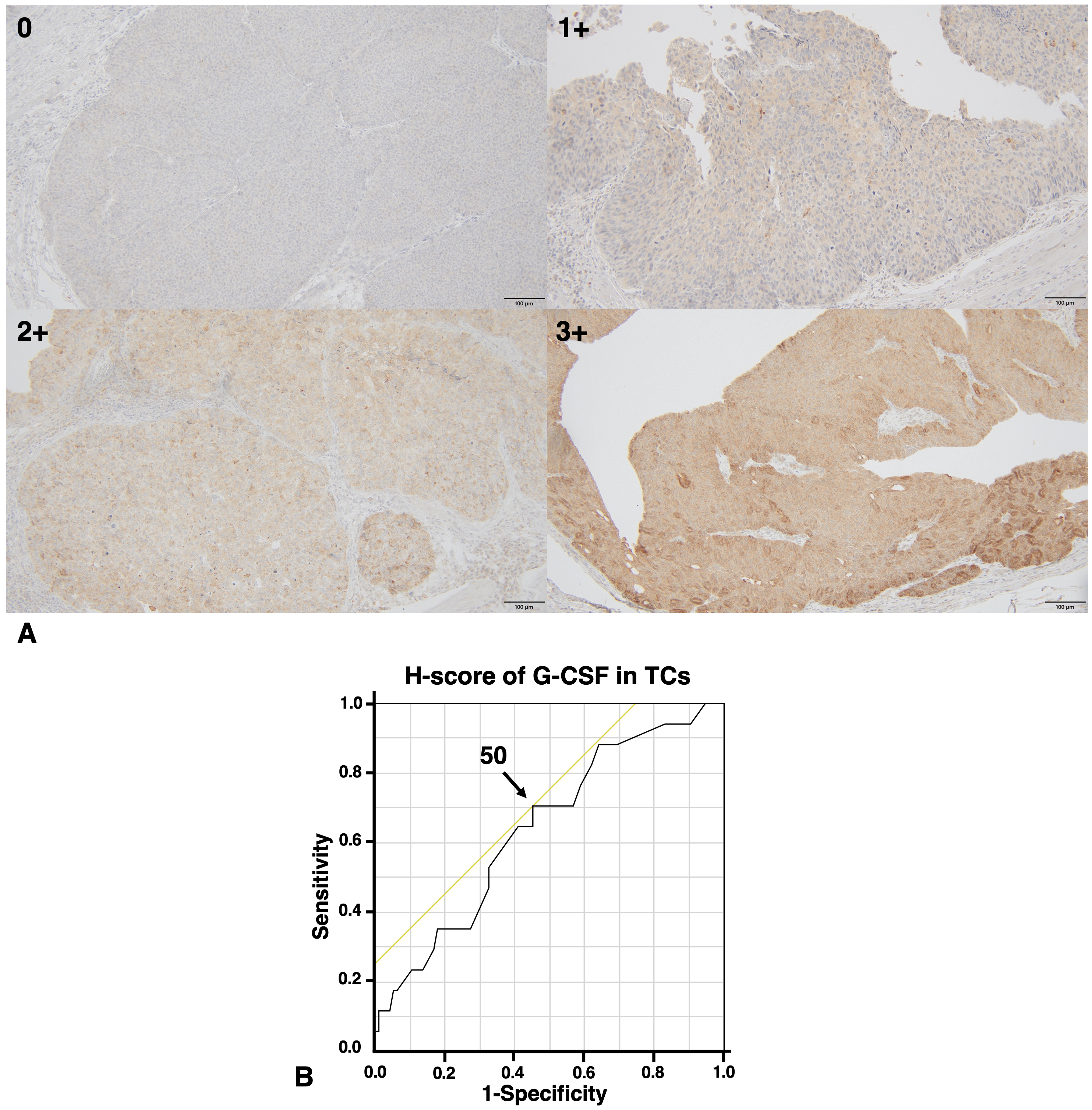

Supplement: Supplementary file 4 — Supplementary Material 4 [file 432_2024_6045_MOESM4_ESM.tiff]
