# Supplementary material for: Distribution and clinicopathological characteristics of G-CSF expression in tumor cells and stromal cells in upper tract urothelial carcinoma
Source: J Cancer Res Clin Oncol. 2024 Dec 30;151(1):18. doi: 10.1007/s00432-024-06045-1 (PMC11685250; doi:10.1007/s00432-024-06045-1)
Supplement: Supplementary file 5 — Supplementary Material 5 [file 432_2024_6045_MOESM5_ESM.docx]

**Table S1. Relationship between G-CSF and blood test results in upper tract urothelial carcinoma.**

|  | G-CSF expression in TCs | | | G-CSF expression in SCs | | |
| --- | --- | --- | --- | --- | --- | --- |
| Factors | Positive | Negative | *P*-value | Positive | Negative | *P*-value |
| White blood cells (Ave ± SD) | 6362 ± 1724 | 6324 ± 2052 | 0.7085 | 6175 ± 1868 | 6387 ± 1876 | 0.5308 |
| Neutrophils (Ave ± SD) | 3060 ± 2033 | 3435 ± 2189 | 0.6594 | 3358 ± 1761 | 3216 ± 2210 | 0.3818 |
| Lymphocytes (Ave ± SD) | 1396 ± 973 | 1540 ± 981 | 0.6447 | 1466 ± 830 | 1470 ± 1020 | 0.6424 |
| NLR (Ave ± SD) | 2.36 ± 0.94 | 2.50 ± 1.12 | 0.6275 | 2.47 ± 0.81 | 2.42 ± 1.11 | 0.5220 |
| Hemoglobin (Ave ± SD) | 12.8 ± 1.71 | 12.9 ± 2.02 | 0.7327 | 13.0 ± 2.21 | 12.9 ± 1.77 | 0.9850 |
| Platelet (Ave ± SD) | 21.60 ± 5.842 | 21.68 ± 6.589 | 0.8174 | 21.31 ± 5.611 | 21.73 ± 6.485 | 0.6990 |
| Albumin (Ave ± SD) | 4.14 ± 0.40 | 4.15 ± 0.36 | 0.9565 | 4.17 ± 0.36 | 4.14 ± 0.39 | 0.7135 |
| Creatinine (Ave ± SD) | 1.02 ± 0.36 | 1.05 ± 0.36 | 0.8380 | 1.04 ± 0.31 | 1.04 ± 0.39 | 0.7556 |
| CRP < 0.3 | 36 (50%) | 50 | 0.6643 | 14 (19%) | 58 | 0.0798 |
| CRP 0.3 ≦ | 13 (37%) | 17 |  | 11 (37%) | 19 |  |
| Abbreviations: G-CSF, Granulocyte-colony stimulating factor; TCs, tumor cells; SCs, stromal cells; Ave, average; SD, standard deviation; NLR, Neutrophil-Lymphocyte Ratio; CRP, C-reactive protein.  *P* values were calculated with Fisher's exact test. | | | | | | |

**Table S2. Comparison of univariate and multivariate Cox proportional hazards analyses of cancer-specific survival in G-CSF expression and other molecules**

|  | Prediction of cancer specific survival | | | |
| --- | --- | --- | --- | --- |
|  | Univariate analysis | | Multivariate analysis | |
|  | HR (95% CI) | *P* | HR (95% CI) | *P* |
| G-CSF in TCs |  |  |  |  |
| Positive | 1 (Reference) | 0.0876 | 1 (Reference) | 0.8982 |
| Negative | 2.48 (0.87-7.05) |  | 1.09 (0.28-4.32) |  |
| G-CSF in SCs |  |  |  |  |
| Positive | 1 (Reference) | **0.0001** | 1 (Reference) | **0.0040** |
| Negative | 6.87 (2.61-19.97) |  | 11.54 (2.09-80.17) |  |
| Ki-67 |  |  |  |  |
| Positive | 1 (Reference) | 0.1618 | 1 (Reference) | 0.8608 |
| Negative | 1.97 (0.72-5.00) |  | 1.14 (0.25-4.83) |  |
| PD-L1 in TCs |  |  |  |  |
| Positive | 1 (Reference) | **0.0079** | 1 (Reference) | 0.1925 |
| Negative | 3.38 (1.42-10.2) |  | 2.84 (0.61-13.28) |  |
| PD-L1 in TILs |  |  |  |  |
| Positive | 1 (Reference) | **0.0473** | 1 (Reference) | 0.6394 |
| Negative | 2.55 (1.01-6,43) |  | 1.48 (0.29-7.42) |  |
| CD8 in TCs |  |  |  |  |
| Positive | 1 (Reference) | 0.9971 | 1 (Reference) | 0.0769 |
| Negative | 1.00 (0.37-2.67) |  | 0.24 (0.05-1.27) |  |
| CD44v9 |  |  |  |  |
| Positive | 1 (Reference) | **0.0231** | 1 (Reference) | **0.0141** |
| Negative | 3.24 (1.13-9.04) |  | 6.01 (1.46-25.22) |  |
| HER2 |  |  |  |  |
| Positive | 1 (Reference) | 0.3425 | 1 (Reference) | 0.4550 |
| Negative | 1.71 (0.56-5.21) |  | 2.01 (0.33-11.92) |  |
| EGFR |  |  |  |  |
| Positive | 1 (Reference) | 0.1733 | 1 (Reference) | 0.8616 |
| Negative | 2.17 (0.61-6.05) |  | 0.86 (0.14-4.30) |  |
| FGFR3 |  |  |  |  |
| Positive | 1 (Reference) | 0.1368 | 1 (Reference) | 0.1745 |
| Negative | 0.39 (0.11-1.35) |  | 0.33 (0.06-1.86) |  |
| p53 |  |  |  |  |
| Positive | 1 (Reference) | 0.1874 | 1 (Reference) | **0.0282** |
| Negative | 1.89 (0.70-4.81) |  | 0.18 (0.03-0.84) |  |
| GATA3 |  |  |  |  |
| Positive | 1 (Reference) | 0.1925 | 1 (Reference) | 0.0609 |
| Negative | 0.48 (0.70-4.81) |  | 7.54 (0.92-95.64) |  |
| CK 5/6 |  |  |  |  |
| Positive | 1 (Reference) | 0.1806 | 1 (Reference) | **0.0103** |
| Negative | 1.95 (0.73-5.21) |  | 10.84 (1.80-65.09) |  |
| Bold values indicate statistical significance (P<0.05).  Abbreviations: HR, hazard ratio; CI, confidence interval; G-CSF, granulocyte-colony stimulating factor; TCs, tumor cells; SCs, stromal cells; PD-L1, programmed death ligand 1; TILs, tumor infiltrating lymphocyte; CD44v9, CD44 variant 9; HER2, human epidermal growth factor receptor type 2; EGFR, epidermal growth factor receptor; FGFR3, fibroblast growth factor receptor; GATA3, GATA binding protein 3; CK 5/6, cytokeratin 5/6. | | | | |
