# Supplementary material for: Distribution and clinicopathological characteristics of G-CSF expression in tumor cells and stromal cells in upper tract urothelial carcinoma
Source: J Cancer Res Clin Oncol. 2024 Dec 30;151(1):18. doi: 10.1007/s00432-024-06045-1 (PMC11685250; doi:10.1007/s00432-024-06045-1)
Supplement: Supplementary file 6 — Supplementary Material 6 [file 432_2024_6045_MOESM6_ESM.docx]

1. **Python cord for ROC curve**

# Importing libraries

import numpy as np

import matplotlib.pyplot as plt

from sklearn.metrics import roc_curve, auc

from sklearn.preprocessing import StandardScaler

from sklearn.linear_model import LogisticRegression

from sklearn.pipeline import make_pipeline

import pandas as pd

# Loading data

data = pd.read_csv('File.csv', index_col=0)

# Delete rows with null values

data.dropna(inplace=True)

# List of explanatory variables

X = data[['GCSF in TCs', 'GCSF in SCs', 'Morphology', 'Grade', 'pT stage', 'ly', 'v']]

# Objective variable

y = data['prognosis']

# Model creation (logistic regression)

classifier = make_pipeline(StandardScaler(), LogisticRegression())

# Calculate ROC curves for each explanatory variable

features = ['GCSF in TCs', 'GCSF in SCs', 'Morphology', 'Grade', 'pT stage', 'ly', 'v']

plt.figure()

# Process each feature in a loop

for feature in features:

X_feature = data[[feature]]

# Predicting poor prognosis using Model

y_score = classifier.fit(X_feature, y).predict_proba(X_feature)[:, 1]

# Calculate ROC curves

fpr, tpr, thresholds = roc_curve(y, y_score)

roc_auc = auc(fpr, tpr)

# Plotting the ROC curve

plt.plot(fpr, tpr, lw=2, label=f'{feature} (AUC = {roc_auc:.2f})')

plt.plot([0, 1], [0, 1], color='gray', lw=2, linestyle='--')

plt.xlim([0.0, 1.0])

plt.ylim([0.0, 1.05])

plt.xlabel('1-Specificity', fontsize=11)

plt.ylabel('Sensitivity', fontsize=11)

plt.legend(loc="lower right")

plt.show()

1. **Python cord for C-index**

# Importing libraries

import pandas as pd

import numpy as np

from lifelines.utils import concordance_index

import matplotlib.pyplot as plt

# Loading data

data = pd.read_csv('File.csv', index_col=0)

# Delete rows with null values

data.dropna(inplace=True)

# Specify explanatory variables for calculating C-index

variables = ['GCSF in TCs', 'GCSF in SCs', 'Morphology', 'Grade', 'pT stage', 'ly', 'v']

# Set time points for calculating C-index for each survival time

time_points = np.linspace(data['Time (D)'].min(), data['Time (D)'].max(), 100)

# For storing results

c_indices_dict = {var: [] for var in variables}

# Calculate C-index for each time point for each explanatory variable

for var in variables:

for t in time_points:

subset_data = data[data['Time (D)'] <= t]

if len(subset_data) > 1 and subset_data['prognosis'].sum() > 0:

c_index = concordance_index(subset_data['Time (D)'], -subset_data[var], subset_data['prognosis'])

c_indices_dict[var].append(c_index)

else: c_indices_dict[var].append(np.nan)

# Calculate average C-index

average_c_indices = {}

for var in variables:

# Calculate average by excluding NaN

valid_c_indices = [ci for ci in c_indices_dict[var] if not np.isnan(ci)]

average_c_indices[var] = np.mean(valid_c_indices)

# Plot for each explanatory variable

plt.figure(figsize=(10, 7))

for var in variables:

avg_c_index = average_c_indices[var]

plt.plot(time_points, c_indices_dict[var], label=f'{var} (Mean C-index: {avg_c_index:.3f})', marker='o')

# Plot settings

plt.xlabel('Time (days)', fontsize=16)

plt.ylabel('Concordance index (C-index)', fontsize=16)

plt.ylim(0.3, 0.85)

plt.axhline(y=0.5, color='gray', linestyle='--', label='Random prediction (C-index = 0.5)')

plt.xticks(fontsize=14)

plt.yticks(fontsize=14)

plt.legend(fontsize=15, loc='lower right')

plt.tight_layout()

plt.show()

1. **Python cord for Uniform Manifold Approximation and Projection (UMAP)**

# Importing libraries

!pip install umap-learn

import numpy as np

import pandas as pd

import urllib.request

import matplotlib.pyplot as plt

%matplotlib inline

import sklearn

from sklearn.preprocessing import StandardScaler

import umap

# Loading data

data = pd.read_csv('File.csv', index_col=0)

# Delete rows with null values

df.dropna(inplace=True)

# Standardization of matrices

dfs = df.iloc[:, 0:].apply(lambda x: (x - x.mean()) / x.std(), axis=0)

dfs.head()

# Running UMAP

umap_model = umap.UMAP(n_neighbors=10, min_dist=0.05, n_components=2, random_state=42)

umap_embedding = umap_model.fit_transform(dfs)

# Convert the UMAP embedding results to a data frame

umap_df = pd.DataFrame(umap_embedding, columns=['UMAP1', 'UMAP2'])

# Specify a column containing category data.

category_col = df.iloc[:, 0]

# Creating a distribution plot

plt.figure(figsize=(6, 6))

plt.scatter(umap_df.iloc[:89, 0], umap_df.iloc[:89, 1], alpha=0.8, c='red', label='faverable')

plt.scatter(umap_df.iloc[89:, 0], umap_df.iloc[89:, 1], alpha=0.8, c='blue', label='poor')

plt.legend(fontsize='17', bbox_to_anchor=(1.05, 1), loc='upper left')

plt.grid()

plt.xlabel("UMAP1")

plt.ylabel("UMAP2")

plt.show()

1. **Python cord for support vector machine (SVM)**

# Importing libraries

import pandas as pd

from sklearn.model_selection import train_test_split

from sklearn.svm import SVC

from sklearn.metrics import accuracy_score, confusion_matrix, classification_report, roc_curve, auc

from sklearn.preprocessing import StandardScaler

import matplotlib.pyplot as plt

# Loading data

df = pd.read_csv('File.csv')

# Delete rows with null values

df.dropna(inplace=True)

# Split features and labels

X = df[['Sex', 'Age', 'Lateralization', 'Location', ' Morphology ', ' Histological classification', 'Histological grade', 'Pathological T stage', ' Concomitant CIS', 'Lympho‐vascular invasion', 'Venous invasion', 'G-CSF in TCs', 'G-CSF in SCs']]

y = df['prognosis']

# Standardization of matrices

scaler = StandardScaler()

X_scaled = scaler.fit_transform(X)

# Split training data and test data

X_train, X_test, y_train, y_test = train_test_split(X_scaled, y, test_size=0.5, random_state=42)

# Building an SVM model (adjusting the class weights)

clf = SVC(kernel='linear', probability=True, class_weight='balanced', random_state=42)

clf.fit(X_train, y_train)

# Predicting with test data

y_pred_test = clf.predict(X_test)

# Predicting with training data

y_pred_train = clf.predict(X_train)

# Model evaluation (test data)

print("Test Data Evaluation")

print("Accuracy:", accuracy_score(y_test, y_pred_test))

print("Confusion Matrix:\n", confusion_matrix(y_test, y_pred_test))

print("Classification Report:\n", classification_report(y_test, y_pred_test))

# Model evaluation (training data)

print("Train Data Evaluation")

print("Accuracy:", accuracy_score(y_train, y_pred_train))

print("Confusion Matrix:\n", confusion_matrix(y_train, y_pred_train))

print("Classification Report:\n", classification_report(y_train, y_pred_train))

# Plot ROC curve (test data)

y_prob_test = clf.predict_proba(X_test)

fpr_test, tpr_test, _ = roc_curve(y_test, y_prob_test[:, 1])

roc_auc_test = auc(fpr_test, tpr_test)

# Plot ROC curve (training data)

y_prob_train = clf.predict_proba(X_train)

fpr_train, tpr_train, _ = roc_curve(y_train, y_prob_train[:, 1])

roc_auc_train = auc(fpr_train, tpr_train)

# Calculate accuracy for Test and Train

accuracy_test = accuracy_score(y_test, y_pred_test)

accuracy_train = accuracy_score(y_train, y_pred_train)

# Plot the ROC curve

plt.figure()

plt.plot(fpr_test, tpr_test, color='darkorange', lw=2,

label='Test ROC curve (AUC = %0.2f, Acuracy = %0.2f)' % (roc_auc_test, accuracy_test))

plt.plot(fpr_train, tpr_train, color='blue', lw=2,

label='Train ROC curve (AUC = %0.2f, Acuracy = %0.2f)' % (roc_auc_train, accuracy_train))

plt.plot([0, 1], [0, 1], color='gray', lw=2, linestyle='--')

plt.xlim([0.0, 1.0])

plt.ylim([0.0, 1.05])

plt.xlabel('1-Specificity', fontsize=11)

plt.ylabel('Sensitivity', fontsize=11)

plt.xticks(fontsize=10)

plt.yticks(fontsize=10)

plt.legend(fontsize=11, loc='lower right')

plt.show()
